# Hydrological Networks and Associated Topographic Variation as Templates for the Spatial Organization of Tropical Forest Vegetation

**DOI:** 10.1371/journal.pone.0076296

**Published:** 2013-10-18

**Authors:** Matteo Detto, Helene C. Muller-Landau, Joseph Mascaro, Gregory P. Asner

**Affiliations:** 1 Smithsonian Tropical Research Institute, Panamá, República de Panamá; 2 Department of Global Ecology, Carnegie Institution for Science, Stanford, California, United States of America; The Ohio State University, United States of America

## Abstract

An understanding of the spatial variability in tropical forest structure and biomass, and the mechanisms that underpin this variability, is critical for designing, interpreting, and upscaling field studies for regional carbon inventories. We investigated the spatial structure of tropical forest vegetation and its relationship to the hydrological network and associated topographic structure across spatial scales of 10–1000 m using high-resolution maps of LiDAR-derived mean canopy profile height (MCH) and elevation for 4930 ha of tropical forest in central Panama. MCH was strongly associated with the hydrological network: canopy height was highest in areas of positive convexity (valleys, depressions) close to channels draining 1 ha or more. Average MCH declined strongly with decreasing convexity (transition to ridges, hilltops) and increasing distance from the nearest channel. Spectral analysis, performed with wavelet decomposition, showed that the variance in MCH had fractal similarity at scales of ∼30–600 m, and was strongly associated with variation in elevation, with peak correlations at scales of ∼250 m. Whereas previous studies of topographic correlates of tropical forest structure conducted analyses at just one or a few spatial grains, our study found that correlations were strongly scale-dependent. Multi-scale analyses of correlations of MCH with slope, aspect, curvature, and Laplacian convexity found that MCH was most strongly related to convexity measured at scales of 20–300 m, a topographic variable that is a good proxy for position with respect to the hydrological network. Overall, our results support the idea that, even in these mesic forests, hydrological networks and associated topographical variation serve as templates upon which vegetation is organized over specific ranges of scales. These findings constitute an important step towards a mechanistic understanding of these patterns, and can guide upscaling and downscaling.

## Introduction

Hydrologic networks are important determinants of the spatial organization of many ecosystems [1]. One prominent feature of the network is its fractal nature, which is common to many other complex systems including atmospheric turbulence, coastlines, tree branches and protein structure. Water and nutrients flow along the network, the network shapes topography over time, and the connectivity and self-organization of the watershed structure directly and indirectly affects vegetation. These influences have long been recognized as central to ecosystem spatial patterns in deserts, savannas, and riparian forests, where water availability has unambiguous effects on plant growth, species distributions, and resulting vegetation structure [2]–[4]. In mesic ecosystems such as tropical forests, the influences of hydrological network and associated topographic variation are more subtle – they do not generally make the difference between vegetation and lack thereof, or between highly distinct vegetation types. However, many studies have shown that tropical forest structure and composition varies between topographically defined habitats such as valleys, slopes and ridges (Table 1). These topographical features differ centrally in their position with respect to the hydrological network, and thus in their exposure to hydrological processes such as erosion, weathering, transport and sedimentation, and in resulting influences on soil properties and soil nutrient availability [5]–[8].

**Table 1:** Studies that have analyzed the relationship between tropical forest structure and topography (in chronological order).

Reference	Sample number x Unit area (spatial grain)	Spatial extent (linear or areal)	Quantitative topographic variables				Descriptive topographic categories	Scale over which topographic variables are assessed	Forest type and location
			elevation	slope	aspect	convexity			
[46]	13×0.6 ha	∼50,000 Km^2^					low hills, slope, ridge, plateau, rocky slope, undulating	Not clear	Mixed Dipterocarp forest, Borneo
[43]	83×0.16 ha	13 ha					ridge, slope, valley, upland valley, riparian valley	Not clear	Wet tropical forest, Puerto Rico
[17]	65×1 ha	1000 Km^2^		X				Not clear	Terra firme wet forest, Central Amazon
[47]	3×4 ha	600 ha		X			terrace/ridgetop, upper slope, mid-slope, base slope/riparian	Not clear	Wet tropical forest, Costa Rica
	1170×0.01 ha								
	18×0.5 ha								
[48]	36×1 ha	unknown	X				valley, mid-slope, upper slope and ridge	Not clear	Mixed Dipterocarp forest, North Borneo
[41]	125×0.4 ha	59 ha					stream, swamp, slope, high plateau, low plateau	Not clear	Lowland moist tropical forest, Central Panama
[49]	3×0.05 ha	unknown					Ridge and lower slope	Not clear	Lower montane forest, Borneo
	2×1 ha								
	×0.2 ha								
[50]	9×0.01 ha	600 m					plateau, slope, valley	Not clear	Lowland evergreen wet forest, Amazon
[51]	72×1 ha	64 Km^2^	X	X				250 m	Terra firme moist tropical forest, Amazon
[52]	1250×0.04 ha	25 ha					ridge and valley	20 m	Wet tropical forest, Ecuador
[25]	2,006,400×0.0025 ha	5,016 ha						Low-elevation, mid-elevation, high-elevation	Tropical forest, Hawaii
[12]	15×1 ha	∼10Km^2^					hilltop, slope, downslope, bottomland	Not clear	Lowland tropical forest, French Guiana
	1×25 ha								
[53]	13×1 ha	1000 Km^2^	X					100 m	Tropical moist forest, Atlantic coast, SE Brazil
[42]	13,959×0.09	1,256 Ha		X				3.4 m	Lowland moist tropical forest, Central Panama
	ha OR								
	1048×1 ha								
[54]	1×2.6 ha	600 Km					valley bottom, lower slope, mid-slope, upper slope, ridge	Not clear	Hill dipterocarp forest, Sumatra
	1×3.3 ha								
	13×4.5 ha								
	1×6.0 ha								
[55]	55×0.018 ha	35 km	X	X				13 m for elevation; not clear for slope	Tropical montane cloud forest, puna, and transition zone, Peru
	11×0.1 ha								
	1 ha/4								
[56]	18×1 ha	∼300 Km	X	X	X			20 m	inhumbane lowland forest, transitional/submontane forest and afromontane forest, Zanzibar -Tanzania
[57]	186,248,800×0.0025 ha	165,617 Km^2^	X	X	X			90 m	Multiple types of tropical forest, Colombian Amazon
[18]	72×1 ha	64 Km^2^		X			valley, slope, plateau	250 m	Terra firme moist forest, Amazon
this study	∼1,960,000×0.0025 ha	49 Km^2^	X	X	X	X		61 scales log-evenly distributed between 10 and 1000 m	Lowland moist tropical forest, Central Panama

The variability of forest structure with its habitat is often studied with topography as a surrogate for environmental spatial heterogeneity. Topographic features such as slope and curvatures can serve as indirect measures of many hydrological processes [9] and digital elevation models (DEM) can be used to infer drainage channels [10]. It is important to note that topography varies over several orders of magnitude, from centimeters (roughness) to hundreds of kilometers (mountain ranges). This extremely wide range of variation is also associated with a multitude of mechanisms that affect different ecological processes in tropical forests. For example, soil chemical properties vary across the landscape in a predictable manner from ridge tops to mid-slopes to riparian valleys [6]. Topography affects the frequency and intensity of many natural disturbances, including landslides, floods, and windthrows [11], [12]. Human land use and anthropogenic disturbance also vary systematically with topography in any given area; for example, farmers often choose flat land and avoid slopes and deep concavities to maximize yields, while leaving trees along watercourses and other inaccessible areas [13], with clear consequences for forest structure [14].

Although many studies have found that tropical forest structure varies in relation to topography (Table 1), these studies share three important shortcomings. First, most of these studies are highly limited in the spatial scales at which they investigated topographic effects, reflecting the dimensions at which the study was conducted, often using plots designed for other purposes. Second, virtually all previous studies assess topographic variables at a single spatial grain, and most do not clarify what that spatial grain is. This is critical because for fractal objects the same type of structures, e.g. ridges, slopes and valley bottoms, reappear almost identically (except for a scaling factor) over and over on a broad range of observational scales. For example, a point on the fork in a stream running down a slope could be located on a very local ridge at 3-m scales, while being within a valley at 30-m scales, and on a slope at 300-m scales. Similarly, the slope computed based on variation in elevation over 3 m is different than the slope computed over 30 m or over 300 m. Third, no previous study in tropical forests has examined the relationship of forest structure with a quantitative measure of landscape convexity – that is, of whether an area is a local depression (valley, swamp) or peak (hilltop, ridge). Yet, the spatial pattern associated with the hydrological network would be expected to follow convexity attributes, and indeed, many of the qualitative categories focus on distinctions involving convexity. For example a map of the concave-upward portions of a DEM can be considered to be an approximation of the drainage network.

What is needed is a multiscale analysis of the relationship between topography and tropical forest structure. Systems that exhibit great variability in the size, shapes and spatial organization of the patterns may be influenced by different processes or combinations of processes [15], [16]. This presents a key challenge for spatial analyses that needs to be addressed quantitatively to permit comparison among studies and assimilation of the results into models. Like topography, tropical forests exhibit large and important spatial variation in structure and biomass at multiple scales. At large scales of 10–1000 km, changes in climate and geological substrate explain variation in old-growth forest type and structure [17]. At intermediate scales of 100 m –10 km, forest structure is strongly associated with variation in water availability, soils, and disturbance [18]. And at local scales of 10–100 m, gap dynamics leads to patchiness related to time since the last large treefall [19].

A fundamental problem in ecology is how to separate variability due to biotic interactions from variability that arises from interaction with the environment. Crown arrangement, tree clustering and gap phase dynamics contribute to create large spatial variability even in a perfectly homogeneous habitat. If the scales of endogenous and exogenous factors are sufficiently separated, spectral decomposition (e.g. Fourier or wavelet transforms) provides a useful tool to systematically analyze the scale-dependent properties of these large spatial datasets, one that is often used in studies of topography [20] and watershed hydrology [21]. The amount of variability present at each scale may reveal fractal similarity in vegetation [22], sometimes attributed to self-organization of ecological systems [23]. However, this fractal similarity could be the result of the association with a self-organized environmental factor, such as the drainage network [24].

**Figure 1:**
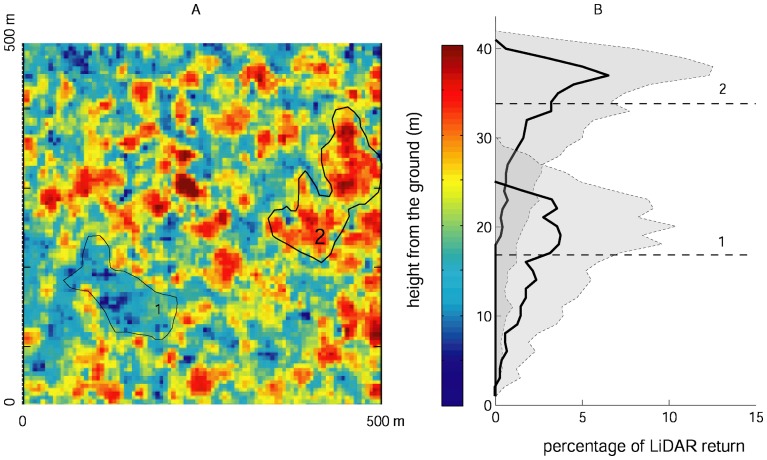
Lidar derived mean profile canopy height. An example of the differences in vertical structure of areas differing in mean canopy height (MCH). A) LiDAR-derived mean canopy height (MCH) for a subset of the study area, polygons marked 1 and 2 delineate two patches of low and high canopy height, respectively. B) Vertical structure of areas 1 and 2 in panel A: horizontal dashed lines show the mean MCH, solid lines depict the median percentage of returns, and shaded areas show the 75^th^ percentile.

Airborne LiDAR offers an excellent tool for investigating spatial variation in tropical forest across different scales. Recent advances in LiDAR (Light Detection and Ranging) techniques have made it possible to monitor forest structure with unprecedented resolution [19], [25]. LiDAR-derived products such as mean canopy profile height (MCH) are well correlated with carbon stocks [19], [26]–[28]. Further, LiDAR not only provides data on the vertical profile of vegetation (Figure 1) but also on ground elevation, enabling the construction of high-resolution digital elevation models (DEM) for extracting hydrological networks analysis of topographic features at the same resolution as the vegetation data.

Here, we quantified the spatial structure of LiDAR-derived MCH in a moist tropical forest in central Panama at multiple scales, and we investigated the association of MCH to the hydrological network extracted from DEM. The study area in Central Panama comprised 4930 ha of mixed old-growth and old secondary forest stands located on a relatively homogeneous geologic formation, where a complex network of small streams discharges into Gatun Lake. These characteristics made the area well-suited for evaluating the influence of the hydrological network and associated topographic features on a relatively large area without confounding variation in lithology, forest type or climate. Our analyses showed a strong association with channels that drain at least 1 ha. This resulted in a scale-dependent correlation between elevation and the vertical structure tropical forest vegetation, with a peak at a characteristic scale of 250 m. This interaction produced fractal similarity of the vegetation patches on a broad range of scales (30–600 m), which we attribute to the self-organization of the hydrological network.

## Materials and Methods

### Study site

**Figure 2:**
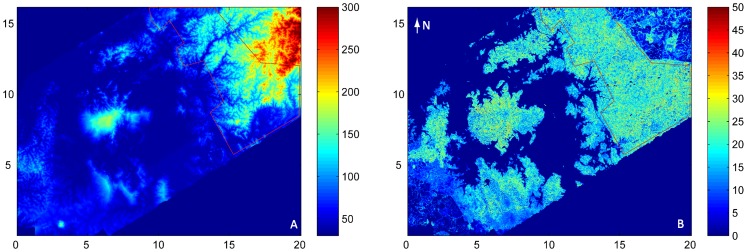
Study area. A) LiDAR-derived DEM and B) LiDAR-derived mean canopy height (MCH) for the study area (red polygon) based on LIDAR data acquired in September 2009. Distances are in km, and heights in m. The large island to the left of the study area is Barro Colorado Island. Note the complex topography of the study area compared to the rest of the region covered by the LiDAR.

The study area is located within Soberanía National Park in Central Panama, east of the Barro Colorado Nature Monument. It consists of a 49.3 km^2^ polygon of mixed old growth and old secondary tropical forest (Figure 2). The climate is tropical moist [29]. Annual precipitation averages approximately 2600 mm, with a pronounced dry season between January and April (a mean of about 3.5 months with <100 mm mo^−1^). Mean annual temperature is 26°C and varies little throughout the year. The soil of the eastern part of Soberanía is a relatively uniform basalt formation; several alluvial sediments with high clay content emerge around the lake (Figure S1).

### LiDAR MCH and DEM

Airborne LiDAR data were acquired for this region in September 2009 by the Carnegie Airborne Observatory (CAO) Alpha Sensor package [30]. The CAO Alpha LiDAR was operated at 2000 m above ground with 1.12-m spot spacing, 30-degree field of view, beam divergence customized to 0.56 mrad, and 50-kHz pulse repetition frequency, for which the aircraft maintained a ground speed ≤95 knots. With these flying parameters, CAO collected continuous laser coverage without gaps between laser spots on the ground. In addition, all flights were planned with 100% repeat coverage (50% overlap between adjacent swaths) and therefore LiDAR pulse density averaged two points per 1.12-m spot.

Canopy three-dimensional structure, as detected by LiDAR, was analyzed by binning discrete LiDAR returns into volumetric pixels (voxels) of 5 m spatial resolution and 1 m vertical resolution, yielding histograms representing the vertical distribution of vegetation in each 5×5 m spatial cell [27], as depicted in Figure 1. These data were further reduced to MCH, which is the volumetric vertical center of the canopy (as opposed to top-of-canopy height), dashed lines in Figure 1. Areas differing in MCH differ systematically in vertical distributions of vegetation (Figure 1).

There are small gaps (<1% total) in both the DEM and MCH maps due to clouds. For the analyses involving a spatial Fourier transform (which requires a continuous field), these gaps were filled via interpolation. For all other analyses, these areas were excluded.

### Multiscale analyses

We used wavelet-based techniques to analyze the spatial structure of MCH and elevation in the study region, and the relationship between the two. These techniques are widely used to study multi-scale processes, because wavelet theory is based on scale-wise decomposition. In particular, wavelets have proven effective in extracting statistical properties of a variety of long-range dependence phenomena, including fractals and other scale-invariant processes, in one or more dimensions [31].

**Figure 3:**
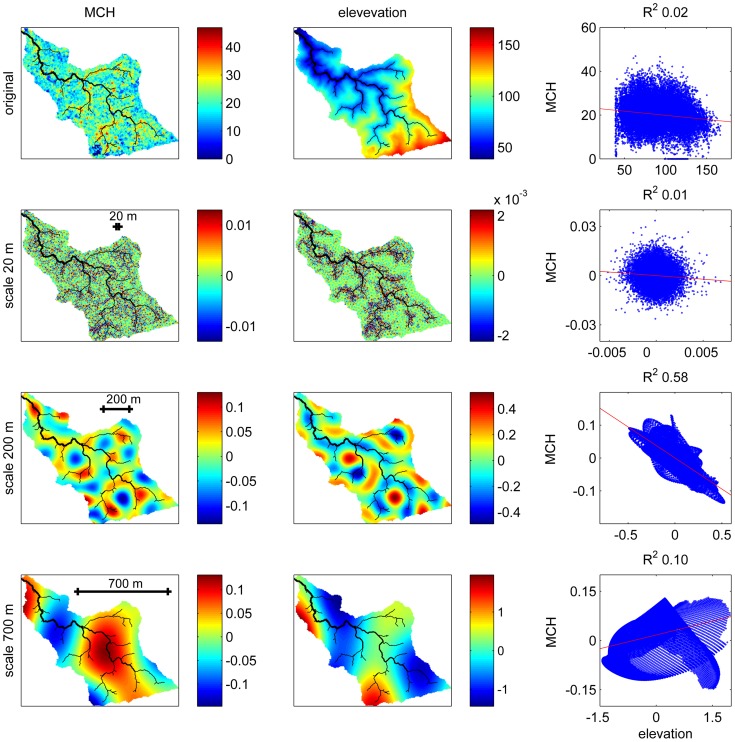
An illustration of how wavelet analysis enables multiscale analyses of bivariate relationships, for a 56 ha watershed extracted from the study area. The top row shows the original, 5-m resolution data for LiDAR-derived mean canopy height (MCH) and elevation, along with a scatterplot showing their bivariate relationship, which is very weak. Wavelet analysis essentially decomposes the total spatial variation in MCH and DEM (top row) into the sum of deviations at different spatial scales, and bivariate wavelet analyses investigates how scale-specific deviations are correlated. Subsequent rows show the wavelet decomposition of MCH and DEM for three arbitrary scales: 20, 200, and 700 m. Scatterplots among these transformed variables (last column) reveal that areas that are locally lower in elevation at scales of ∼200 m tend to have higher biomass (3^rd^ row), while at smaller or larger scales the correlation is weaker and may even be in the opposite direction. For reference, the drainage network (minimum drainage area 0.25 ha) is shown in black on the maps, and the ordinary linear regression lines in red on the scatterplots.

Here we focus on the variance of a 2-D spatial process, studied using wavelet decomposition (see appendix S1 for details). To illustrate how this approach works, Figure 3 shows maps of MCH and elevation for a 56 ha watershed extracted from the study area, and their wavelet decompositions at three arbitrary scales (20, 200, and 700 m). Essentially, wavelet analysis decomposes the total variation into the sum of deviations at different scales. Thus the map of wavelet coefficients at the 700 m scale shows only the broader pattern of deviations, while the map at the 20 m scale shows only fine-grained variation deviating from broader patterns. The scatter plots of the relationships between elevation and MCH, and their scale-specific deviations as captured by the wavelet decomposition, show that at the 200 m scale, deviations in MCH and in DEM are strongly negative correlated, while at smaller or larger scales the correlation is less significant and can even have the opposite sign. Because the relationships between the variables differ with scale, analyses should be carried out systematically at all possible scales, from the smallest scale allowed by the resolution of the remote sensing product to the maximum scale enabled by the spatial extent of the study area (before edge effects become dominant).

We first conducted univariate wavelet analyses for elevation and MCH, examining the contributions of variation at different spatial scales to total variation in each variable. Specifically, we calculated the wavelet spectrum; that is, the wavelet variance as function of scale *s*. A plot of the wavelet spectrum depicts the contribution to the total spatial variability of structures or patterns with a typical scale comparable to *s*. A peak in the spectrum means that patterns of a characteristic scale are dominant. A scale-invariant process exhibits no peaks in the wavelet spectrum; its wavelet variance is a power function of scale (*∼s^a^),* and it appears linearly increasing on a log-log representation.

We examined whether elevation and MCH had anisotropic (directional) patterns by performing spectral analyses with an anisotropic Morlet wavelet. Examples of anisotropic patterns are waves in the sea. The results were evaluated using circular plots of the wavelet variance, with scale as the radial coordinate and angle as the azimuthal coordinate [32]. If there is more variation in some directions than others, this indicates the presence of anisotropy in the spatial patterns (which may exist for all scales or for just a narrow range of scales or none at all). The anisotropic analyses were carried out on circular subsets of the studied area to avoid edge effects.

To evaluate the bivariate relationship between elevation and MCH at multiple scales, we calculated the wavelet co-spectrum and wavelet coherence between them. The wavelet co-spectrum is a measure of covariance among spatial processes at different scales, while the wavelet coherence is a measure of correlation. The wavelet coherence is analogous to an R^2^ value, and ranges from 0 to 1. We calculated confidence intervals for the null hypothesis of no correlation (*H_0_*: *wch*  = 0) using Monte Carlo methods. Specifically, we used the iterative amplitude adjusted Fourier transform (IAAFT) to randomize one of the processes (in our case MCH) in a way that preserves the same probability density function as the original template and also preserves its structure and in particular the second-order moments (the spectral density or the autocorrelation function) [33], [34]. For an example, see figure S2.

### Topographic and hydrological variables

The drainage network was extracted from the DEM using the D8 algorithm [10], [35] implemented in ArcGIS (ESRI, v10). We qualitatively assessed the relationship of MCH to the drainage network by visually inspecting maps that overlaid MCH and the drainage network at different sub-basin scales. We quantified the relationship of flow distance to the drainage network with wavelet transformed MCH. Here too we took a multiscale approach: channels were defined as those that drain a minimum area *A* and we varied *A* from 25 m^2^ to 50,000 m^2^. We examined the Pearson correlation between wavelet transformed MCH and distance to the drainage network as a function of both MCH scale and minimum area *A* (with minimum area *A* essentially a scale metric for the hydrological network).

**Figure 4:**
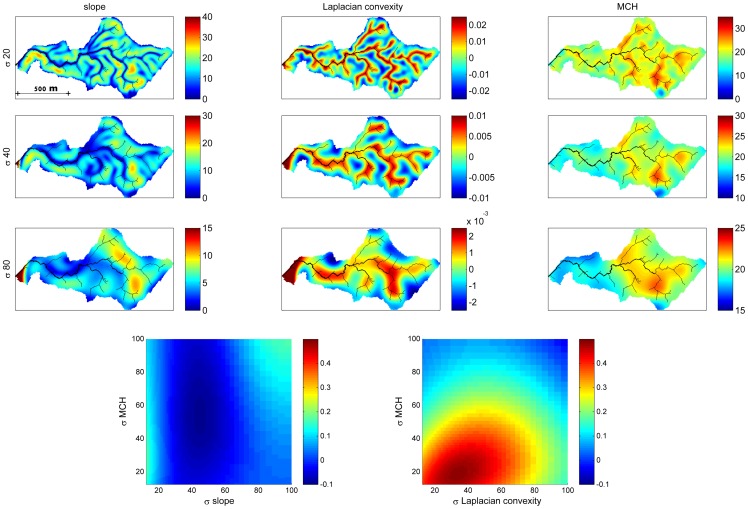
An illustration of the effect of smoothing at different scales on the spatial patterns of slope, Laplacian convexity and MCH (top), and on the correlation of MCH with slope and convexity, for a 58.6 ha watershed extracted from the study area. Smoothing was done with a Gaussian smoothing filter of standard deviation 20, 40 and 80(minimum drainage area 0.25 ha) is shown in black on the maps for reference. The correlation coefficient between MCH and slope or convexity at different combinations of smoothing scales is shown in the bottom panels.

We computed slope, aspect, Laplacian convexity, and various measures of curvature using linear and nonlinear combinations of first and second order derivatives of the smoothed DEM (Table 2), for smoothing at multiple scales [21]. Smoothing was performed using Gaussian kernels for 61 different scales ranging log-evenly between 2.5 and 1250 m. We smoothed at multiple scales because the slope and other topographic variables take different values depending on the degree of smoothing, which essentially gives the scale at which the variables are calculated. This is illustrated in Figure 4, which depicts maps of slope, convexity, and MCH under smoothing at three different scales, for a 60 ha watershed extracted from the study area. For no smoothing or only small-scale smoothing, the topographic variables reflect very local features at the resolution of the DEM, while for large scale smoothing they reflect larger scale features. As shown in this example, MCH may be related to topographic variables calculated at some scales, but not others, and thus analyses should be conducted based on smoothing at all possible scales. Note that the wavelet transform of the DEM with a Mexican Hat wavelet is identical (except for a normalization factor) to the Laplacian convexity obtained after smoothing the elevation map with a Gaussian kernel [21]. We evaluated pairwise correlations of untransformed MCH with each topographic variable at each scale. Specifically, for the noncircular variables, we calculated Pearson correlation coefficients; for the circular variables of slope and aspect, we calculated linear-circular correlations [36]. We repeated these correlations for wavelet transformed MCH for wavelets at the scale for which the coherence of MCH and elevation was maximized.

**Table 2:** Topographic variables computed, with their formulas.

Topographic variable	Formula [58]	Hydrological significance (adapted from Moore et al. 1991)
*slope*	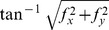	Overland and subsurface flow velocity and runoff rate
*aspect*	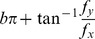	Solar irradiation
*tangent curvature*	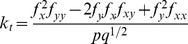	Erosion/deposition rate
*contour curvature*	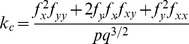	Converging/diverging flow, soil water content
*mean curvature*	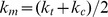	concavity/convexity
Laplacian convexity*	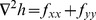	valley bottom (positive values) vs. ridge top (negative values)

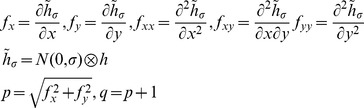
 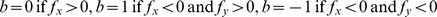
* The Laplacian convexity obtained after smoothing the elevation map with a Gaussian kernel is identical to the wavelet transform of the DEM with a Mexican Hat wavelet, except for a normalization factor [21].

## Results

**Figure 5:**
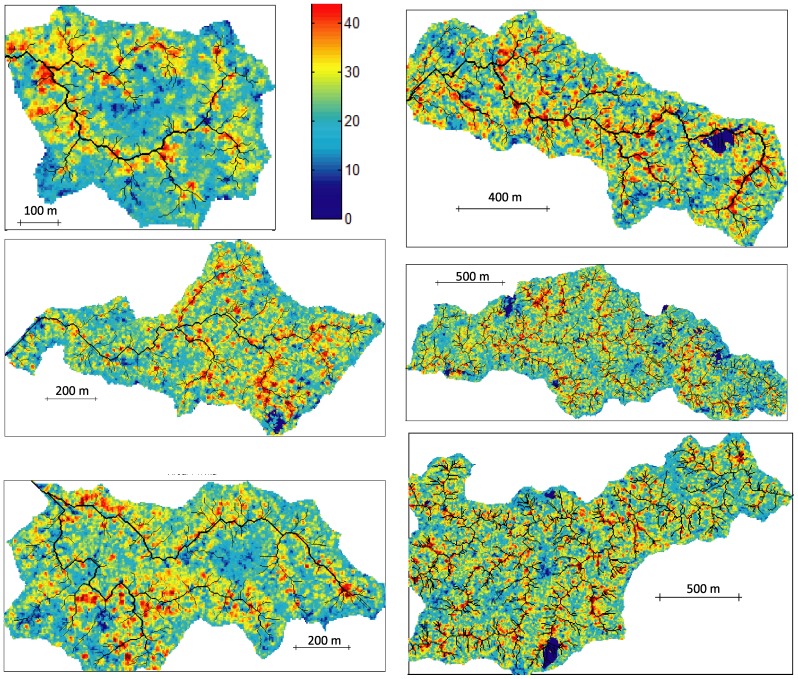
Maps of drainage networks and LiDAR-derived MCH for a collection of watersheds in Soberanía National Park (Panama). Drainage networks (minimum drainage area 0.25 ha) are delineated with black lines, and colors indicate MCH (in m). Dark blue areas are cloud coverage.

Visual inspection of maps superimposing MCH and the drainage networks of individual watersheds showed that tall trees (high MCH) tend to occur along channels (Figure 5). This is more immediately apparent in higher-resolution figures (the smaller basins in Figure 5). The lower-resolution figures of larger basins provide evidence that this behavior is general.

**Figure 6:**
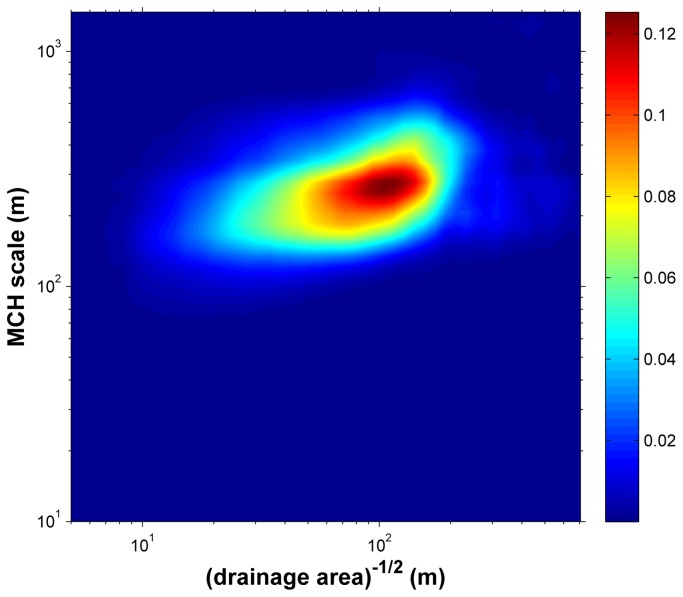
R^2^ between flow distance to channel as function of minimum drainage area and wavelet transform MCH as function of scale.

We quantified this visual interpretation by analyzing the correlation of MCH with the flow distance to the drainage network (Figure 6). Two scale dependences can be found in this analysis. The first is the minimum flow accumulation area that defines a channel. The second is related to the size of the vegetation patchiness corresponding to the scale of the wavelet transform. Correlations between wavelet transformed MCH and the map of distance from closest channels for different accumulation areas and scales were maximized for proximities to channels that drain at least 1 ha of forest and are associated with variation in MCH at scales of ∼230 m.

**Figure 7:**
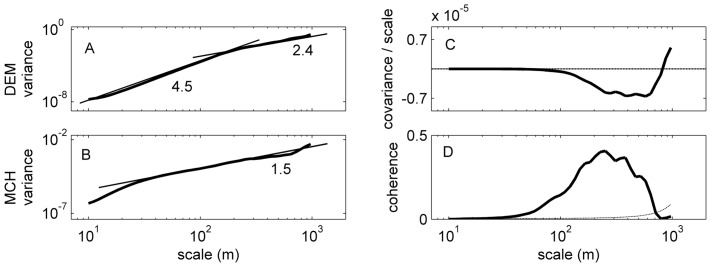
Wavelet spectra of elevation (A) and LIDAR-derived mean canopy height (B) in the entire study area (delineated in Figure 2). Tangent lines and their slopes are shown for reference. (C) Normalized wavelet cospectrum between MCH and elevation (solid line); the area between the spectrum and the zero line (dashed line) is proportional to the total covariance. (D) Wavelet coherence between MCH and elevation (solid line) as a function of scale, compared with the 95% confidence interval for the null hypothesis of no correlation computed with 1000 IAAFT surrogates of the MCH map (dashed line).

Spectral analysis of the elevation and MCH data revealed that both have self-similar patterns over a wide range of scales, with some notable deviations. The wavelet spectrum of elevation increased smoothly and approximately linearly on log-log scales, with a change in slope around 200 m (Figure 7A). At small scales, the logarithmic slope of the scaling was steep, with power law exponent 4.5. At larger scales, the exponent is 2.4, which was closer to the average values for continents of 2.09 [20]. The wavelet spectrum of MCH also showed a clear linear scaling over a wide range of distances, with a scaling exponent of 1.5 between 30 and 500 m (Figure 7B). At distances less than 30 m, the spectrum increased more steeply.

The wavelet covariance showed that MCH and elevation co-vary negatively for scales of 100–1000 m (Figure 5C). The coherence analysis (a spectral analog of R^2^) revealed a broad range of scales (30–700 m) with strong correlation with a peak of ∼0.4 at approximately 250 m (Figure 7D), a value consistent with the analysis in Figure 6. This result was extremely robust, as is clear from comparison with the 95% confidence level for the null hypothesis of no correlation (dashed line in Figure 5D).

**Figure 8:**
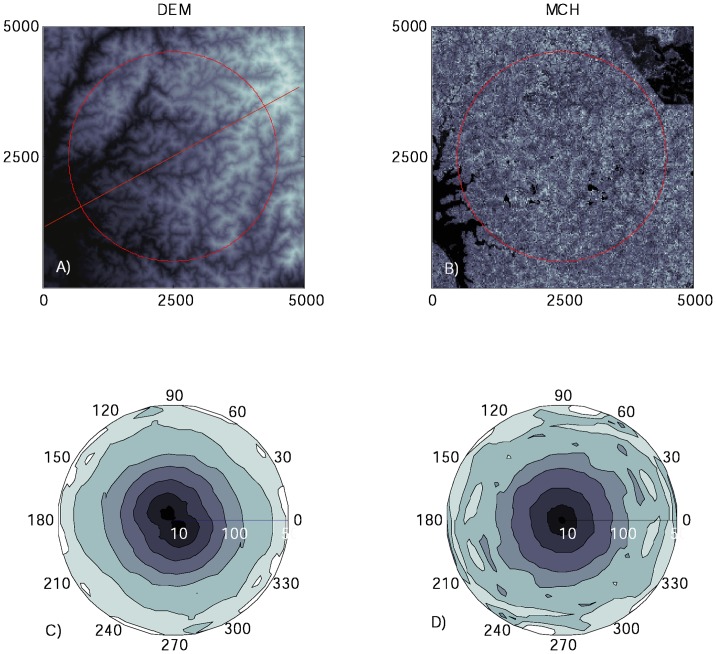
Analyses of directional patterns in the DEM (A) and MCH (B) maps, conducted using anisotropic wavelet analysis, for a 1250-ha circular subset of the study area (red). Panels C and D depict the wavelet variance of DEM and MCH, respectively, as a function of the scale (radial coordinate) and the angle of orientation of the wavelet (azimuthal coordinate) (North  = 0). The straight dashed line in panel A depicts the direction of maximum variance of the DEM.

The anisotropic spectral analysis of the largest possible circular subset of the study area (Figure 8A,B) revealed that the DEM has a small asymmetry in the NE-SW axis, as indicated by the elliptical shape of the contour lines (Figure 8C). This suggested that the morphological structures are generally stretched and oriented towards the direction of main drainage flow into Gatun Lake. In contrast, the MCH did not show any evidence of anisotropy, as the contour lines were approximately circular for all scales (Figure 8D). Results were very similar for other circular subsets.

**Figure 9:**
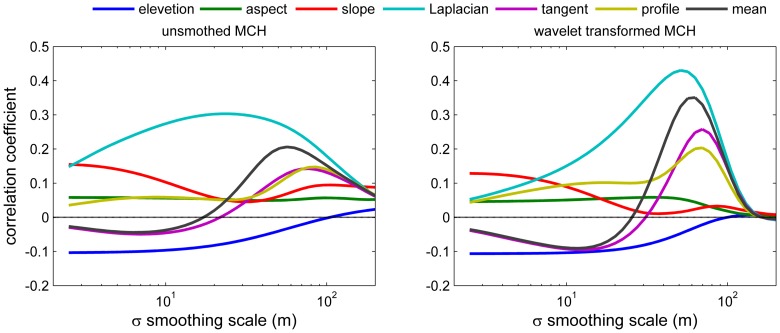
Top) Correlation coefficients between unsmoothed MCH and smoothed topographic variables (defined in Table 2), as a function of the smoothing scale. For slope and aspect a linear-circular correlation is used [36]; for the other variables, Pearson's correlation. Bottom) same as above, but for wavelet transformed MCH at 250 m scale.

All the topographic variables were correlated with MCH at some scales, with the Laplacian convexity showing the greatest correlation (Figure 9). Laplacian convexity essentially captures whether an area is low or high relative to the surroundings: positive values are associated with depressions and valleys, and negative values with peaks or ridges. The degree of correlation varied strongly with the smoothing scale for every variable except aspect. The strongest correlation with MCH was found for the convexity calculated with a Gaussian filter of 20 m, corresponding to a smoothing scale of ∼100 m [21]. For this case, the Pearson correlation was 0.3, meaning this variable alone explained 9% of the total variance of MCH among 5×5 m pixels (top Figure 9), that is the resolution of this LiDAR product. For small smoothing scales below 10 m, slope was more highly correlated with MCH than was Laplacian convexity. Note that the Laplacian convexity is strongly associated with distance to drainage channels, with higher values indicating greater proximity to channels as shown in the example in Figure 4. Qualitatively similar results are found using the wavelet transformed MCH at 250 m scale (bottom Figure 9), which corresponds to the peak of the coherence analysis in Figure 7. The correlations are stronger in this case, peaking at 0.43, because only the patterns at this scale are considered, while small scale (e.g. crown, gap-phase) and large scale variability are filtered out.

## Discussion

### Scale-dependence and self-similarity

The spatial distribution of vegetation reflects both exogenous influences of habitat and endogenous factors such as dispersal limitation and gap-phase dynamics, with different processes contributing to patterns at different scales. Here, we demonstrated the utility of spectral analyses, which decompose variation across scales, for investigating spatial variation in mean canopy height, and its relationship with the hydrological network and associated topographic attributes. Our analyses showed that variation in mean canopy height at scales of ∼100–400 m is strongly related to proximity to channels that drain a minimum of 1 ha of forest land, and to topographical convexity calculated at these scales. Overall, there was a scale dependent relationship between topography and MCH with a peak at 250 m.

We found that the spatial variance of MCH has fractal similarity over scales of 30–600 m, a new and important finding. This fractal similarity can be explained by the association of MCH variation at these scales with fractal environmental structure, specifically the hydrological network. Previous studies of LIDAR derived indices in tropical forests also found self-similar scaling relationships, albeit at smaller scales, where they may not necessarily be associated with topography [19], [37].

The self-similarity in MCH provides a critical basis for upscaling and downscaling. For many natural patterns, such as the species-area curve, scale-invariance is present only for a limited range of scales; the broader the range, the wider the spectrum of possible applications. The fractal similarity of MCH in the range 30–600 m is potentially extremely useful for up-scaling or down-scaling remote sensing products using forest inventory plots, as the scales of remote sensing footprints and forest inventory plots are often quite different [38]. For example, the application of non-linear models at coarser scales [39] requires knowledge of sub-grid variability [40]. Information on how the variance changes with scales can also be used to optimize the size of ground-based plots for remote sensing calibration products. And finally, knowledge of how the variance changes with scale enables meaningful comparisons of studies carried out at different scales.

### Hydrological network, topography and tropical forest structure

Our results expand on previous findings of the influence of topographic variation for tropical forest structure by identifying the topographic variables and scales at which relationships are strongest, and by clearly illustrating their link with the hydrological network. Previous studies computed topographic variables at just one scale, and often did not state what that scale was. Yet we demonstrated that the relationship of tropical forest structure to topographic variables depends strongly on the scale at which those variables are computed. Approximately half of previous studies used qualitative topographic categories rather than quantitative topographic variables, limiting the potential to generalize from such studies. Of the studies that did use quantitative variables, the most common variable examined was slope, then elevation and aspect, but not convexity, despite the fact that most of the qualitative categories are centrally about convexity. We found that convexity explained much more variation in MCH than did slope, with convexity calculated with a 100 m smoothing proving the single best predictor of MCH among all topographic variables.

Overall, we found a strong pattern of larger canopy height in areas of positive convexity (valleys, depressions) close to channels of the drainage network (Figures 5, 6). This is broadly consistent with conventional wisdom among foresters, which states that in drier areas forests are taller in valleys, while in wet habitats forests are taller on ridges. However, an explicit connection to the hydrological network, and the quantification of minimum drainage area necessary to create this link, has never been made in moist tropical forests. Previous studies conducted in the nearby island of Barro Colorado, employing different methods, have failed to detect this association. Chave *et al.* (2003) found lower biomass in riparian areas and higher biomass on slopes within a 50 ha plot, while Mascaro *et al.* (2011a) found that mean canopy height increased with the steepness of slopes across the island as a whole. This highlights the utility of extensive remote sensing data and multi-scale analyses in providing a new perspective on even comparatively well-known tropical sites.

Identification of the scales at which topography exerts the greatest influence on forest structure and of convexity as the variable that best captures these influences constitutes an important step towards a mechanistic understanding of these patterns. Although water and/or nutrient availability appear the most obvious explanation of these patterns because they are directly influenced by hydrological processes [9], other mechanisms cannot be ruled out. For example, convexity is also related to natural disturbance rates, with hilltops more susceptible to windthrow and landslides, the most frequent causes of large gap formation in these type of forests [43], [44]. Convexity is also strongly related to anthropogenic land clearing in this area, as trees tend to be left standing around watercourses and seasonal channels (Figure S3). Our identification of convexity as the key variable lays the groundwork for future studies testing these alternative mechanisms through investigation of spatial variation in tree demographic rates, water availability, nutrient availability, and the history of human disturbance over the focal scales identified here.

### Conclusions and recommendations for future work

The relationship between tropical forest structure, topography and the hydrological network is fundamentally scale-dependent, and thus can only be properly understood with multi-scale analyses. Our analyses provide the first clear evidence of the scale-dependent linkage of the hydrological network and associated topographic attributes to patterns of vegetation structure in closed-canopy tropical forests. Regardless of the mechanism, the association of MCH with convexity, and of convexity with the drainage channels, essentially means that the hydrological network can be considered a template for the spatial organization of the forest.

Future work should evaluate the generality of our results, and aim to elucidate the mechanisms that underlie them. Studies should specifically investigate how the association with hydrological networks and topography varies as function of hydrological zone, geological formation, climate and disturbance regimes, and how spatial variance in MCH scales in these other forests. Our study focused on a distinct region of the river system which is called, following Schumn [45], the *production zone*. In the *transfer zone* and in the *deposition zone*, these patterns are likely to differ or even disappear. Additional spatial data on water availability, soil fertility, disturbance frequency, land use history, tree demography, and species composition, among others, could illuminate which mechanisms are most important in determining the relationship between MCH and the drainage network at different scales, and potentially different sites. Disentangling the roles of different factors is likely to be challenging because many factors covary spatially; study design, and especially appropriate choice of scales, is thus critical. Well-designed studies of this kind have the potential to greatly improve our understanding of tropical forest structure, and our ability to project the responses of tropical forests to anthropogenic global change.

## Supporting Information

Appendix S1 **Multiscale analyses.**
(DOCX)

Figure S1 (DOCX)

Figure S2 (DOCX)

Figure S3 (DOCX)
