# Supplementary material for: Hydrological Networks and Associated Topographic Variation as Templates for the Spatial Organization of Tropical Forest Vegetation
Source: PLoS One. 2013 Oct 18;8(10):e76296. doi: 10.1371/journal.pone.0076296 (PMC3799763; doi:10.1371/journal.pone.0076296)
Supplement: Appendix S1 — Multiscale analyses. (DOCX) [file pone.0076296.s001.docx]

**Appendix S1: Multiscale analyses**

We used wavelet-based techniques to analyze the spatial structure of MCH and elevation in the study region, and the relationship between the two. These techniques have been widely adopted to study multi-scale processes, because wavelet theory is based on scale-wise decomposition. In particular, wavelets have proven effective in extracting statistical properties of a variety of long-range dependence phenomena, including fractals and other scale-invariant processes, in one or more dimensions (Heneghan, Lowen, & Teich, 1996). Here we focus on the variance of a 2-D spatial process, studied using wavelet decomposition.

Given a spatial process f(**x**),with the vector **x** = [x, y] denoting Cartesian coordinates, the wavelet coefficients W_f_ (**b**,s,_ _) are defined as

 (S1)

where is the complex conjugate of the scaled, translated and rotated mother wavelet, s the scale at which the transform is applied, **b** the translation vector, and *θ* the angle of orientation of the anisotropic wavelet. Usually the convolution integral is computed in the Fourier transform to take advantage of the fast Fourier routine:

 (S2)

where ˄ indicates 2-D Fourier transform and the vector **k** = [k_x_, k_y_] denotes the angular frequency space. One of the most commonly used wavelets is the Morlet, which has a Fourier transform given by

 (S3)

where c_φ_ is a constant to ensure unit energy, k_0_ is a shape parameter, *ε* is the anisotropic ratio and are the rotated angular frequencies with rotation matrix

.

If the problem is isotropic, it is possible to express the Morlet in a simplified isotropic form and remove the dependence on *θ_ _*:

 (S4)

The functions *φ* are essentially band-pass filters whose properties depend on the associated scaling parameter, s. They enable a focus on features of the process whose detail matches their scale parameters, i.e., broad features for large *s* and fine features for small *s* (Praveen Kumar & Foufoula-Georgiou, 1997). The wavelet variance or spectrum, S_ff_ (s,*θ_ _*), is computed as:

 (S5)

integrated over a sub-domain *A* which can be considered weakly stationary (i.e. the first and second order moments are spatially homogeneous).

We calculated the wavelet spectrum for elevation and for MCH. A plot of the wavelet spectrum – that is, the wavelet variance as function of scale *s* – depicts the contribution to the total spatial variability by structures or patterns with a typical scale comparable to s. A peak in the spectrum means that patterns of a characteristic scale are dominant. A scale-invariant process exhibits no peaks in the wavelet spectrum; its wavelet variance is a power function of scale (S_ff_ ~s^a^), meaning S_ff_ appears linear on a log-log representation.

We examined whether elevation and MCH had anisotropic patterns by performing spectral analyses with an anisotropic Morlet with * * varying. The results were evaluated using circular plots of the wavelet variance, with scale as the radial coordinate and angle as the azimuthal coordinate (P Kumar, 1995). If there is more energy in some directions than others, this indicates the presence of anisotropy in the spatial patterns (which may exist for a limited number of scales only). The anisotropic analyses were carried out on circular subsets of the studied area to avoid edge effects.

We calculated the wavelet co-spectrum and wavelet coherence between elevation and MCH, to examine how they are related at different scales. The wavelet co-spectrum, C_fg_(s,*θ_ _*), between two spatial variables f(**x**) and g(**x**) shows how two processes co-vary as functions of scale, and is defined as

 (S6)

Combining wavelet spectra and cospectra we compute the wavelet coherence, which is a measure of correlation among spatial processes at different scales and different anisotropic angles:

 (S7)

The wavelet coherence is analogous to an R^2^ value, and ranges from 0 to 1. We calculated confidence intervals for the null hypothesis of no correlation (H_0_: wch = 0) using Monte Carlo methods.
Specifically, we used the iterative amplitude adjusted Fourier transform (IAAFT) to randomize one of the processes (in our case MCH) in a way that preserves the same probability density function as the original template and also preserves its structure and in particular the second-order moments (the spectral density or the autocorrelation function) (Schreiber & Schmitz, 2000; Venema, 2006). We generated 1000 IAAFT data surrogates for MCH in a rectangular subset (for an example, see supplemental materials S3) and calculated the wavelet coherence with elevation for each surrogate. We then compared the coherence of true MCH and elevation with the 95% confidence interval generated from these surrogates.

*Topographic and hydrological variables*

We calculated maps of slope, aspect, and various measures of curvature (Table 1) based on elevation maps smoothed at multiple scales (Lashermes, Foufoula-Georgiou, & Dietrich, 2007). Smoothing was performed using Gaussian kernels for 61 different scales ranging log-evenly between 2.5 and 1250 m. We smoothed at multiple scales because the slope and other topographic variables take different values depending on the degree of smoothing, which essentially gives the scale at which the variables are calculated, and MCH may be related to the value based on smoothing at some scales but not others. (For no smoothing or only small-scale smoothing, the topographic variables reflect very local features, while for large scale smoothing they reflect larger scale features.) These topographic variables were computed using linear and nonlinear combinations of first and second order derivatives of the smoothed DEM (Table 1). High-order derivatives of the DEM were computed from smoothed elevation maps where *h* is the elevation, *H* some smoothing function (here a Gaussian). By the properties of the convolution, the *n*x*m*-order derivative can be computed as

 (S8)

where *N*(***x****,*) is a Gaussian kernel with variance *σ^2^*. The convolution is again efficiently computed using the FFT routine.

Spectral properties of elevation derivatives (or any linear combination thereof) follow directly from elevation spectral properties. For example, the Lapacian is the sum of the second derivatives in the *x* and *y* directions, and thus essentially reflects the local convexity of the landscape. Derivatives of a Gaussian are wavelets (the second derivative is also called the Mexican Hat wavelet), which in Fourier space are expressed, for any order *m* and *n* as

 (S9)

Hence, the wavelet transform of the DEM with a Mexican Hat is identical (except for a normalization factor) to the Lapacian obtained after smoothing the elevation map with a Gaussian kernel. Spectral properties of non-linear combinations of derivatives are less straightforward.

We evaluated pairwise correlations of MCH with each topographic variable at each scale. Specifically, for the noncircular variables, we calculated Pearson’s correlation coefficients. For the circular variables of slope and aspect, we calculated linear-circular correlations (Mardia, 1976).

The drainage network was extracted from the DEM using the D8 algorithm (Jenson & Domingue, 1988; O’Callaghan & Mark, 1984) implemented in ArcGIS (ESRI, v10). Channels were defined as those that drain an area of 250 m^2^ or more. We qualitatively assessed the relationship of MCH to the drainage network by visually inspecting maps that overlaid MCH and the drainage network at different sub-basin scales. We then calculated maps of flow distance from nearest stream, defined as the distance that an imaginary water particle travels to reach the closest channel, and we correlated this distance with MCH across the landscape. Finally, we computed the average value of MCH conditioned upon the distance to the nearest channel for the real dataset, and compared these with 95% confidence intervals from the surrogates generated using IAAFT. All analyses were done in Matlab v7 (R2010a).

References

Heneghan, C., Lowen, S., & Teich, M. (1996). Two-dimensional fractional Brownian motion: wavelet analysis and synthesis. *Proceedings of the IEEE Southwest Symposium on Image Analysis and Interpretation, 1996.* (pp. 213–217).

Jenson, S., & Domingue, J. (1988). Extracting topographic structure from digital elevation data for geographic information system analysis. *Photogrammetric engineering and remote sensing*, *54*(11), 1593–1600.

Kumar, P. (1995). A wavelet based methodology for scale-space anisotropic analysis. *Geophysical research letters*, *22*(20), 2777–2780. doi:10.1029/95GL02934

Kumar, Praveen, & Foufoula-Georgiou, E. (1997). Wavelet analysis for geophysical applications. *Reviews of Geophysics*, *35*(4), 385–412. doi:10.1029/97RG00427

Lashermes, B., Foufoula-Georgiou, E., & Dietrich, W. E. (2007). Channel network extraction from high resolution topography using wavelets. *Geophysical Research Letters*, *34*(23), 1–6. doi:10.1029/2007GL031140

Mardia, K. (1976). Linear-circular correlation and rhythmometry. *Biometrika*, *63*, 403–405.

O’Callaghan, J., & Mark, D. (1984). The extraction of drainage networks from digital elevation data. *Computer vision, graphics, and image*, *28*(3), 323–344.

Schreiber, T., & Schmitz, A. (2000). Surrogate time series. *Physica D: Nonlinear Phenomena*, *142*(3-4), 346–382. doi:10.1016/S0167-2789(00)00043-9

Venema, V. (2006). Surrogate cloud fields generated with the iterative amplitude adapted Fourier transform algorithm. *Tellus*, *58A*, 104–120.

%%wavelet variance Matlab code,

% input

% s: mxn matrix

%

% output

% E: vector wavelet variance

[m,n]=size(s);  % matrix size

dj=0.15;        % scale discretization parameter

k0=8;           % Morlet shape factor

J1=50;          % number of scales

Ff=4*pi/(k0+sqrt(4+k0^2));  % Fourier factor

scale = 2*2.^((0:J1)*dj); %define scale array

f = fft2(s);                % 2-D fast Fourier transform

FS(1,:) = abs(f(:)).^2/m/n; % magnitude square

pulsx = 2*pi/n*[0:floor((n-1)/2)  floor((1-n)/2):-1];

pulsy = 2*pi/m*[0:floor((m-1)/2)  floor((1-m)/2):-1];

[kx,ky] = meshgrid(pulsx,pulsy); % frequencies space matrices

k=sqrt(kx.^2+ky.^2);

KS=(k(:)*scale/Ff);

H=exp(-(KS-k0).^2);

E(:,1)=FS*H./sum(H);

%%%%%%%%%%%%%%%%%%%%%%%%%%%%%%%%

%%wavelet co-variance Matlab code s1 and s2 input mxn matrices

[m,n]=size(s1); % matrix size

dj=0.15;        % scale discretization parameter

k0=8;           % Morlet shape factor

J1=50;          % number of scales

Ff=4*pi/(k0+sqrt(4+k0^2));  % Fourier factor

scale = 2*2.^((0:J1)*dj); %define scale array

f1 = fft2(s1);                % 2-D fast Fourier transform

f2 = fft2(s2);                % 2-D fast Fourier transform

FS(1,:) = f1.*conj(f2)/m/n; % magnitude square

pulsx = 2*pi/n*[0:floor((n-1)/2)  floor((1-n)/2):-1];

pulsy = 2*pi/m*[0:floor((m-1)/2)  floor((1-m)/2):-1];

[kx,ky] = meshgrid(pulsx,pulsy); % frequencies space matrices

k=sqrt(kx.^2+ky.^2);

KS=(k(:)*scale/Ff);

H=exp(-(KS-k0).^2);

E(:,1)=FS*H./sum(H);
