# Supplementary material for: Hydrological Networks and Associated Topographic Variation as Templates for the Spatial Organization of Tropical Forest Vegetation
Source: PLoS One. 2013 Oct 18;8(10):e76296. doi: 10.1371/journal.pone.0076296 (PMC3799763; doi:10.1371/journal.pone.0076296)
Supplement: Figure S1 — (DOCX) [file pone.0076296.s002.docx]

**Supplementary figure S1**


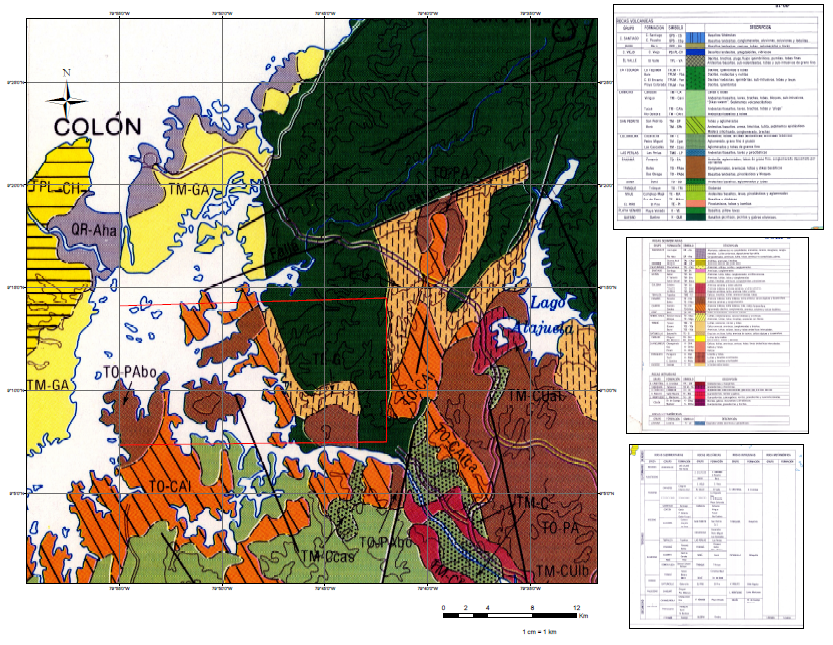


**Figure S1**. Geological map of Soberania National Park and surrounding areas. (original map and compilation from Woodring, 1957)

W. P. Woodring. 1957. Geology and Paleontology of Canal Zone and adjoining parts of Panama. United States Geological Survey Professional Paper 306(A).
