# Supplementary material for: Hydrological Networks and Associated Topographic Variation as Templates for the Spatial Organization of Tropical Forest Vegetation
Source: PLoS One. 2013 Oct 18;8(10):e76296. doi: 10.1371/journal.pone.0076296 (PMC3799763; doi:10.1371/journal.pone.0076296)
Supplement: Figure S2 — (DOCX) [file pone.0076296.s003.docx]

**Supplementary figure S2**

**Figure S2**. A map of MCH and the drainage network in a 180-ha subset of the study area (left), together with an example of an IAAFT surrogate for the same area. Note that the surrogate preserves the spatial structure in MCH observed in the original, but the correlation with the drainage network (black lines) is lost.
