# Supplementary material for: Hydrological Networks and Associated Topographic Variation as Templates for the Spatial Organization of Tropical Forest Vegetation
Source: PLoS One. 2013 Oct 18;8(10):e76296. doi: 10.1371/journal.pone.0076296 (PMC3799763; doi:10.1371/journal.pone.0076296)
Supplement: Figure S3 — (DOCX) [file pone.0076296.s004.docx]

**Supplementary figure S3**

**Figure S3A**. Map of MCH and the drainage network in a part of the Agua Salud ecosystem services project. (See <http://www.ctfs.si.edu/aguasalud/>). Distances and heights are in meters.


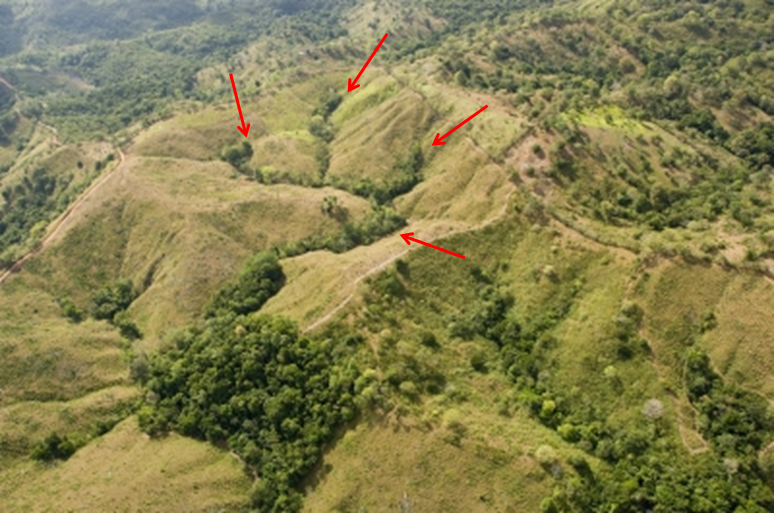


**Figure S3B**. Aerial photograph of part of the Agua Salud study area (source <http://www.ctfs.si.edu/aguasalud/>). The arrows indicate vegetation structure clearly associated with the drainage network.

Source: [http://www.ctfs.si.edu](http://www.ctfs.si.edu/)
